# Rosette-forming glioneuronal tumours: two case reports and a review of the literature

**DOI:** 10.1259/bjrcr.20210125

**Published:** 2021-11-26

**Authors:** Xiao-dan Huang, Han-wen Zhang, Yu-ning Feng, Yi Lei, Fan Lin

**Affiliations:** 1 Department of Radiology, The First Affiliated Hospital of Shenzhen University, Health Science Center, Shenzhen Second People’s Hospital, Shenzhen, China

## Abstract

Rosette-forming glioneuronal tumour (RGNT) is a rare central nervous system (CNS) neoplasm that typically arises in the fourth ventricle. It is even more uncommon to arise outside the midline. In this paper, we report two cases of RGNT: one located in the fourth ventricle (a typical site), and the other in the right cerebellar hemisphere (a rare site). Both cases were misdiagnosed on imaging, and the results were inconsistent with the pathological diagnosis. The aim of the article is to deepen medical practitioners’ understanding of RGNT by learning from these two cases, summarising cases located in the cerebellar hemispheres and systematically reviewing RGNT.

## Summary

Rosette-forming glioneuronal tumour (RGNT) is a very rare but benign tumour of the central nervous system (CNS), first proposed by Komori in 2002.^1^ Due to its connection with the fourth ventricle, it was named “rosette-forming glioneuronal tumour of the fourth ventricle” in 2007 based on the unique structure of neural and glial components.^2^ As RGNT arising outside the fourth ventricle has been successively reported, which was renamed “rosette-forming glioneuronal tumours” and classified as a mixed neuron-glial tumour of WHO Class I in the 2016 version of the CNS classification.^3^ Currently, its origin is unclear. It is generally believed by researchers that it originates from the pluripotential subependymal cells (the periventricular germinal matrix) near the midline.^4^ It usually occurs in the midline and typically in the fourth ventricle and the cerebellar vermis.^5^ Here, we report two cases of RGNTs, with one being a more typical example and one being a more rare example.

## Clinical presentation

### Case 1

**Figure 1.**
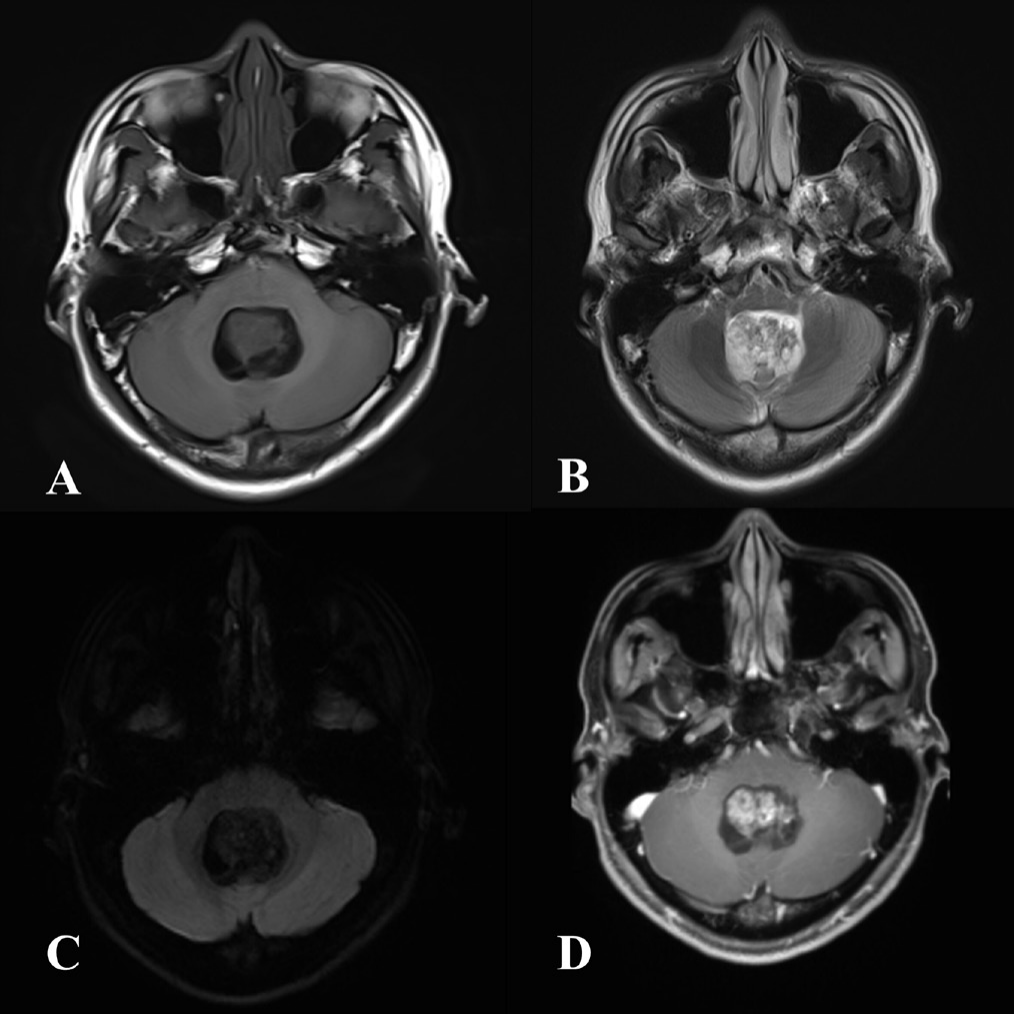
A 27-year-old male patient with intermittent posterior occipital distension and heaviness. MR examination: A. isohypointensity on axial *T*_1_ weighted imaging; B. isohyperintensity on axial *T*_2_ weighted imaging; C. the lesion appears hypointense on DWI; D. heterogeneous enhancement on the *T*_1_ weighted image with gadolinium. DWI, diffusion-weighted imaging.

The patient was a 27-year-old male. 8 months ago, he presented with intermittent posterior occipital distension and heaviness of no obvious cause. Later, the patient’s headache symptoms worsened. The rest of the examination showed no significant abnormalities. Brain CT suggested the presence of tumorous lesions of the fourth ventricle. MRI examination in our hospital (Figure 1) showed a solid cystic occupancy in the patient’s fourth ventricle with a size of approximately 26 × 19 × 33 mm. The lesion grew downward along the fourth ventricles. The brainstem was compressed, and the supratentorial ventricles were expanded. Overall, the lesion showed a mixed signal on *T*_1_ weighted imaging (*T*_1_WI); on *T*_2_ weighted imaging (*T*_2_WI), the lesion showed a hyperintense signal. There was no obvious diffusion limitation on diffusion-weighted imaging (DWI). An enhanced scan showed heterogeneous and obvious enhancement. Imaging indicated a diagnosis of ventricular meningioma.

The patient underwent fourth ventricle space-occupying lesion resection under general anaesthesia, and the operation progressed smoothly.

**Figure 2.**
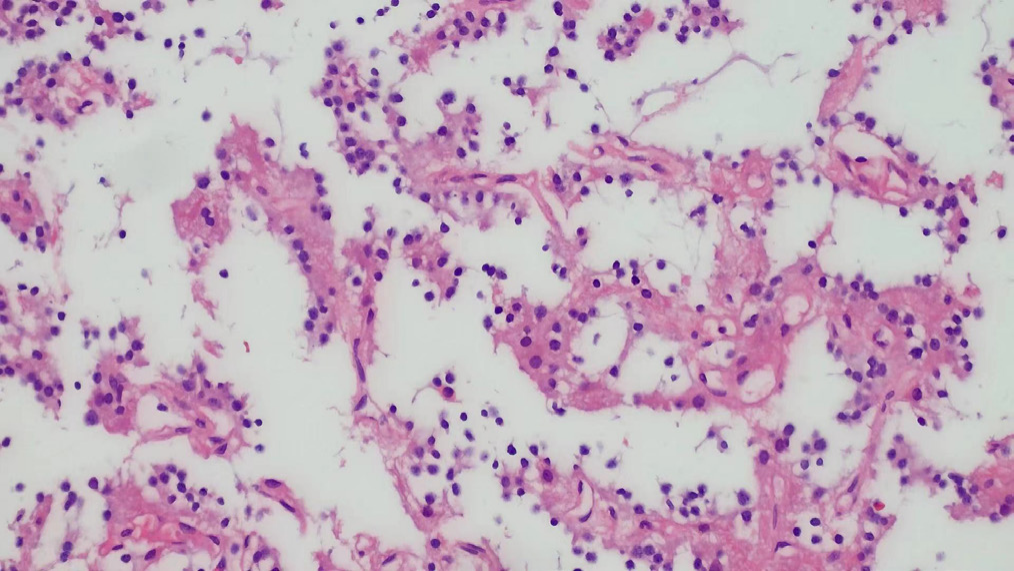
H&E, ×200 shows tumour cells of relatively consistent size distributed in clusters or sheets. Some of them showed daisy-shaped clusters of perineural felts. The nucleus was centred in some areas, and the cytoplasm was translucent and showed an oligodendritic pattern.

Pathological results and light microscopy (Figure 2): the tumour cells were homogeneously distributed and uniform in size, with round nuclei and partial perinuclear cytoplasmic vacuolation.

Immunohistochemical results revealed the following: GFAP(+), Ki67 approximately 1%(+), P53(+), S100(+), Olig-2(+), H3K27Me3(+), Syn(+), NeuN(-), CgA(-), Braf(-).

Fluorescence *in situ* hybridization (FISH) results: no co-deletion of 1p/19q in tumour cells; IDH1 and IDH2 were wild type; and MGMT methylation was negative. In addition, there was no BRAF-KIAA1549 gene fusion. The final pathological results indicated “RGNT” in the fourth ventricle.

### Case 2

**Figure 3.**
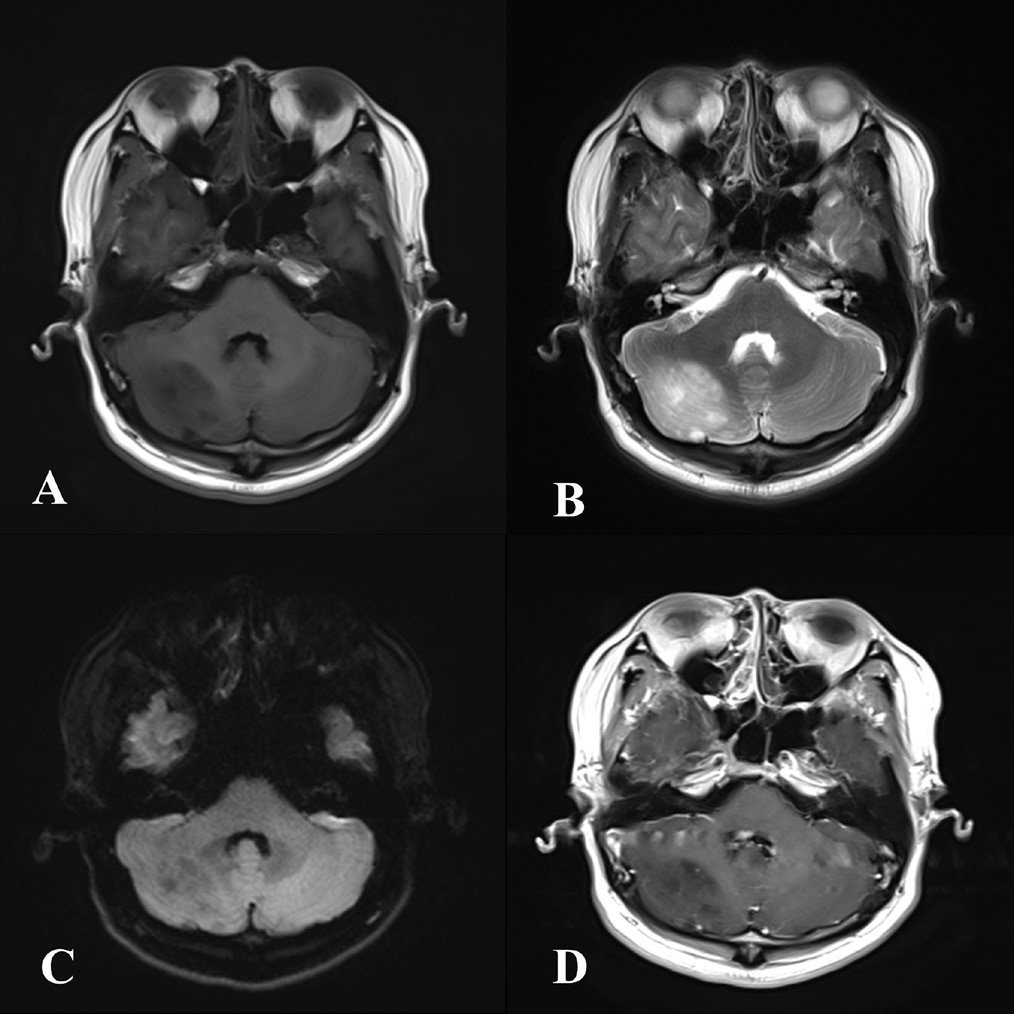
A 31-year-old male patient with no symptoms. MR examination: A. heterogeneous slightly hypointense signal on axial *T*_1_ weighted imaging; B. the lesion appears hyperintense on axial *T*_2_ weighted imaging, and the normal striation of the cerebellar hemisphere can be seen; C. hypointensity on DWI; D. no enhancement of the lesion was found on post-contrast *T*_1_ weighted MRI. DWI, diffusion-weighted imaging.

A 31-year-old male was found to have an occupying lesion in the right cerebellar hemisphere on physical examination. No abnormality was found on examination. MRI examination in our hospital (Figure 3) showed space-occupying lesions in the right cerebellar hemisphere. The lesion was mostly hypointense on *T*_1_ weighted images, *T*_2_ weighted images showed predominantly hyperintense signal. There was no obvious enhancement after contrast injection. It was misdiagnosed as astrocytoma on imaging.

The patient underwent right cerebellar space-occupying lesion resection under general anaesthesia, and the operation progressed smoothly.

**Figure 4.**
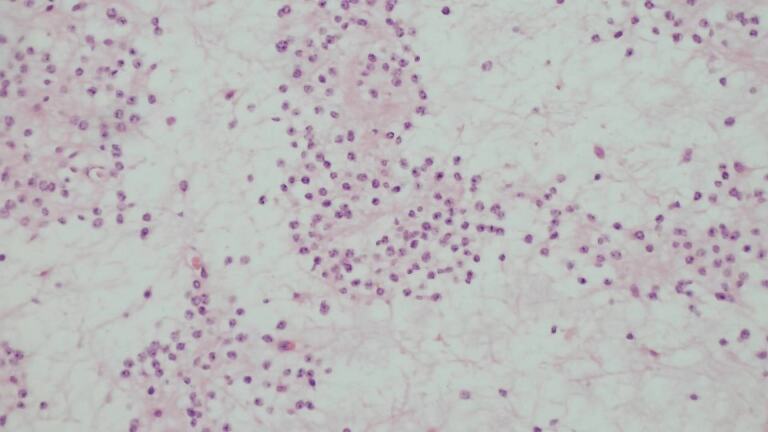
H&E, ×200 revealed tumour cells of a relatively uniform size and clustered distribution. Some of them showed perineural felt daisy-like clusters and interstitial partially mucinous-like degeneration

Pathological results and light microscopy (Figure 4) showed that the tumour cells were visible.

Immunohistochemical results revealed the following: CD34(−), Olig-2(+), GFAP(+), IDH1(−), NeuN(−), Syn(+), NF(+), Ki67 index <1%(+), S100(+), P53 slightly(+), CgA(−), H3K27M(−), H3K27Me3(−). FISH results revealed codeletion of 1p/19q(−), and IDH1 and IDH2 were wild type. The pathological results were consistent with RGNT-like manifestations.

## Discussion

RGNT is a rare benign primary tumour of the nervous system. It is mostly found in young people with a mean age of 29.8 years.^6^ It is slightly more common in females than in males.^5^ It frequently arises near the midline, most commonly in the fourth ventricle and cerebellar vermis. It can also occur in the pineal region, third ventricle, optic chiasm and spinal cord.^5,6^ The clinical manifestations of RGNTs are associated with the location of the lesion.^6,7^ Symptoms include headache, cerebellar ataxia, visual disturbances, nausea, and vomiting as well as vertigo.^6^

Of the two cases reported here, in Case 1, all information including site, clinical symptoms, imaging and pathological manifestations, was consistent with typical RGNT. In contrast, Case 2 occurred at an extremely rare site (cerebellar hemisphere), and all clinical data were unremarkable. Interestingly, in this patient, the imaging data were different from previous tumorigenic lesions, as we could see normal striation in the cerebellar hemisphere. Therefore, we searched the literature and found a total of only six reports on RGNT involving the cerebellar hemispheres and summarised them in a table^8–13^ (TableTable 1). Including this case reported here, no obvious difference was seen in the male to female ratio among these seven reports. We found four patients (57.1%) who presented with headache. On MRI, all the lesions had low signal intensity on *T*_1_WI and high signal intensity on *T*_2_WI. Enhanced scans vary in performance. Ultimately, all seven patients were treated by surgery.

**Table 1.** Cases of rosette-forming glioneuronal tumor of the cerebellar hemisphere

Author	Publication date	Sex	Symptoms	Location	T1WI	T2WI	MRI enhanced scan	Treatment
Preusser et al.^8^	2003	M	Not obvious	Cerebellar vermis, fourth ventricle	Low	High	Ring enhancement	Resection
Luan et al.^9^	2010	F	Headache	And right cerebellar hemisphere	Low	High	Point enhancement	Gross resection
Matyja et al.^10^	2010	F	Headache, nausea,	Right cerebellar hemisphere	Low	High	Ring enhancement	Partial excision
García et al.^11^	2014	M	Balance disorder	Cerebellar vermis and part of the left side	Low	High	Ring enhancement	Surgery radiotherapy and chemotherapy
Beuriat et al.^12^	2015	F	Headache	Cerebellar hemisphere	Low	High	No enhancement	Subtotal resection
Kwon et al.^13^	2019	M	Headache, dizziness, and nausea	Spinal cord, cerebellar hemisphere and	Low	High	Uneven enhancement	Gross resection

Influenced by the components of the tumour, RGNTs tend to appear as cystic and solid occupancies on imaging. The density of solid lesions on CT is lower than that of normal brain parenchyma. Calcifications are visible internally.^14^ On MRI, the solid component of the tumour appears to have a hypo- or isosignal intensity on *T*_1_WI and high signal intensity on *T*_2_WI. Because of the low malignancy of the tumour and the lack of dense cell distribution, DWI usually has no significant diffusion limitation.^7,14^ Enhanced scans may show a special feature called “the green bell pepper sign”.^14^ The mass shows ring-like enhancement, the mucus component in the centre shows low signal intensity, and the surrounding tumour cells show low or no enhancement. The imaging presentation of two cases was consistent with the above. Due to the loose distribution of tumour tissue, there was no enhancement in Case 2. Inside the tumour, we could even see the normal striation of the cerebellar hemisphere, which is quite rare. In addition, several cases of spontaneous disappearance of RGNT enhancement have been reported without intervention. They have occurred as a manifestation of degenerative and inflammatory changes within the tumour affecting the permeability of the blood–brain barrier.^15^

The imaging of RGNTs lacks specificity, and pathological examination is the gold-standard for diagnosis. In histopathological studies, RGNTs exhibit a characteristic biphasic structure: neuronal and glial cells coexist in a mixture. As previously described for RGNTs, the nerve cells are consistent in size and uniformly arranged.

They form a rosette or pseudorosette structure around the blood vessels,^5^ and the centre of the rosettes are eosinophilic neuropil cores. The neuropil cores display strong immunoreactivity for synaptophysin, and the cytoplasmic protrusions express neuron-specific enolase (NSE). The glial component is similar to pilocytic astrocytoma. Immunoreactivity of glial fibrillary acidic protein (GFAP) and S100 is shown in the glial component. A low Ki67 index and a typically negative P53 are consistent with the diagnosis of low-grade tumours in RGNT. IDH-1/2 mutations may also be present in genetic testing. In addition, Philipp Sievers et al^16^ reported the possibility of FGFR1 and PIK3CA co-mutations in RGNT.

### Differential diagnosis

Based on the absence of specificity of clinical manifestations and radiographic features of RGNT, the diagnosis can typically be confirmed only by post-operative pathology. The primary differential diagnosis of RGNT is as follows:

1. Pilocytic astrocytoma (PA): prevalent in children, it is mostly a cystic tumour with wall nodules. It has a similar presentation to RGNT on a plain scan, but wall nodules, not the cyst wall, are significantly strengthened.
2. Dysembrioplastic neuroepithelial tumour (DNET): DNET frequently occurs in children and adolescents, most of whom have a long history of drug-refractory epilepsy. The most common site of onset is the temporal lobe, followed by the frontal lobe. The imaging is also similar to RGNT, and most of them do not have significant enhancement on enhanced scans. It is often associated with cortical dysplasia. Mainly, there is no typical rosette structure of DNET.
3. Ependymoma: this tumour originates from the intraventricular ventricular epithelium. It is frequently seen in children under 5 years of age, and the fourth ventricle is considered the most common site. The tumour is often seen as a necrotic cystic lesion, and MRI often shows mixed signals with moderate enhancement on enhanced scans. The tumour may grow along the ventricular canal towards the Luschka or Magendie foramen of the fourth ventricle, forming a “cast” structure of the ventricle.
4. Central neurocytoma is usually located near the Monro foramen in the lateral ventricle. The signal of the solid portion is equal to the brain tissue, and the cystic portion is uncommon. Calcification and flow-through are characteristic of this tumour. 
5. Medulloblastoma: tt is highly malignant and has a high incidence in the age group of 4 to 8 years. The tumour mostly occurs in the cerebellar vermis and easily protrudes into the fourth ventricle. It has a long T1 signal and long T2 signal on MRI. DWI shows significantly limited diffusion, and the enhanced scan often shows more homogenous enhancement, so it is easy to distinguish from RGNT.
6. Diffuse midline glioma: As the most specific type of molecular typing in the 2016 version of WHO CNS tumours, it is also prevalent at midline sites but has a high degree of malignancy (WHO IV). It is diagnosed based on H3K27M positivity. On imaging, it is generally challenging to distinguish RGNT from RGNT. H3K27Me3 is antagonistic to H3K27M, and patients with positive H3K27Me3 generally have a relatively high prognosis. Both of our cases were positive for H3K27Me3, so theoretically, the prognosis for RGNT is generally better.

### Treatment

The current treatment of choice for RGNTs is surgery. Presently, gross total resection (GTR) is mainly recommended, and subtotal resection (STR) is preferred as an alternative.^6^ Despite the slow progression and good prognosis of RGNT, cases of dissemination to the soft meninges, cerebrospinal fluid, and even malignancy have been gradually reported.^5,11^ RGNT progresses slowly and has a good prognosis. In cases of soft meningeal and cerebrospinal fluid dissemination or even malignancy,^5,11^ adjuvant treatment options such as concurrent chemoradiotherapy can usually be added.^6^

In conclusion, RGNT is a rare benign tumour of the neurological system. In addition to the common sites in the midline region, such as the fourth ventricle and the cerebellar vermis, it can also occur in bilateral cerebellar hemispheres. Case 1 is a typical case. Case 2 has a rare location and radiographic manifestation, not only arising in the right cerebellar hemisphere but also exhibiting cerebellar striation within the tumour. We hope to improve the understanding of RGNT among medical professionals by discussing these two cases.

## Learning points

- RGNT is a rare type of brain tumour that can occur at sites other than the midline.
- On enhanced scans, RGNT may show a special feature called “the green bell pepper sign”.
- Theoretically, patients diagnosed with RGNT with positive H3K27Me3 generally have a relatively high prognosis.
- The greatest difference between RGNT and other CNS tumours is the unique rosette structure of the pathology.
